# Supplementary material for: Mechanisms governing the pioneering and redistribution capabilities of the non-classical pioneer PU.1
Source: Nat Commun. 2020 Jan 21;11:402. doi: 10.1038/s41467-019-13960-2 (PMC6972792; doi:10.1038/s41467-019-13960-2)
Supplement: Supplementary file 7 — Source data [file 41467_2019_13960_MOESM7_ESM.zip › Source_Data/Figure6/Figure6B_MotifScanOutput/FLI1_PU.1/homerResults/motif26.similar.html]

motif26

## Information for motif26

A
G
C
T
A
C
G
T
G
A
C
T
C
G
A
T
C
A
G
T
C
G
T
A
A
C
G
T
T
A
G
C
C
G
A
T
  
Reverse Opposite:  

G
C
T
A
A
T
C
G
T
G
C
A
A
C
G
T
G
T
C
A
C
G
T
A
C
T
G
A
C
G
T
A
T
C
G
A
  

|  |  |
| --- | --- |
| p-value: | 1e-52 |
| log p-value: | -1.200e+02 |
| Information Content per bp: | 1.671 |
| Number of Target Sequences with motif | 959.0 |
| Percentage of Target Sequences with motif | 9.64% |
| Number of Background Sequences with motif | 1919.6 |
| Percentage of Background Sequences with motif | 5.26% |
| Average Position of motif in Targets | 101.6 +/- 54.0bp |
| Average Position of motif in Background | 100.8 +/- 67.4bp |
| Strand Bias (log2 ratio + to - strand density) | 0.1 |
| Multiplicity (# of sites on avg that occur together) | 1.06 |
| Motif File: | file (matrix) reverse opposite |

### Similar de novo motifs found

|  |  |  |  |  |  |  |  |
| --- | --- | --- | --- | --- | --- | --- | --- |
| Rank | Match Score | Redundant Motif | P-value | log P-value | % of Targets | % of Background | Motif file |
| 1 | 0.836 | A T C G A G T C A G C T A C G T C G A T A C T G C G T A C A G T T A C G C G A T | 1e-50 | -117.312751 | 5.36% | 2.26% | motif file (matrix) |
| 2 | 0.881 | C G T A A T G C G T C A A C G T G T A C C G T A C G T A C G T A T C A G T C G A T G C A | 1e-39 | -91.420229 | 3.58% | 1.38% | motif file (matrix) |
| 3 | 0.834 | A C G T A C G T A C G T A C T G C G T A A C G T A T C G A C G T | 1e-39 | -90.539549 | 4.51% | 2.00% | motif file (matrix) |
